# Optimisation of food grade mixed surfactant‐based l‐ascorbic acid nanoemulsions using response surface methodology

**DOI:** 10.1049/nbt2.12014

**Published:** 2021-02-10

**Authors:** Tahir Mehmood

**Affiliations:** ^1^ Institute of Food and Nutritional Sciences PMAS‐Arid Agriculture University Rawalpindi Pakistan

## Abstract

Co‐surfactant free l‐ascorbic acid (LAA) nanoemulsions were prepared using mixed surfactants (Soya lecithin and Tween 80). Response surface methodology (RSM) was used to optimise the emulsifying conditions for LAA nanoemulsions. The effects of water proportion (6%–14% w/w), homogenisation pressure (80–160 MPa), surfactant concentrations (4%–12% w/w) and laa concentration (0.5–1.3 w/w) on responses (size of droplets and nanoemulsion stability) were investigated. RSM results showed that the values of responses can be successfully predicted through second‐order polynomial model. The coefficients of determinations for droplet size and nanoemulsion stability were 0.9375 and 0.9027, respectively. The optimum preparation conditions for l‐LAA nanoemulsion were 9.04% water proportion, 114.48 MPa homogenisation pressure, 7.36% surfactant concentration and 1.09% LAA concentration. At the end of one month storage study, the retention of LAA in optimised nanoemulsions stored at 4°C and 25°C were 74.4% and 66.7%, respectively. These results may provide valuable contributions for food and pharmaceutical industry to develop delivery system for food additives and nutraceutical components.

## 1 INTRODUCTION

Food products fortification and use of different additives during preparation of food products are increasingly used in the food industry [1]. Food additives found numerous applications where hydrophilic active components (water soluble vitamins, nutraceuticals, flavours and antioxidants) are incorporated into organic media (lipid phase) to make them suitable for oral consumption [2, 3, 4, 5]. The most convenient way to achieve this objective is to incorporate hydrophilic food additives in W/O nanoemulsions to protect them from degradation [6]. Due to their smaller droplet size (20–200 nm diameter), these nanoemulsions enhanced bioavailability, kinetic stability, bioefficacy and solubility of hydrophilic compounds [7]. Previously, few studies were carried out on l‐ascorbic acid (LAA) emulsions [4, 8], but no study was carried out on LAA‐based nanoemulsions. Hence, present study was designed to prepare LAA nanoemulsion.

Nanoemulsions can be prepared either using mixed surfactants or single surfactant. Mixed surfactants perform better than single surfactant because they improve the stability of nanoemulsion by increasing the solubility of encapsulated compound and producing smaller droplet size [6, 9]. The smaller size of the droplet is produced due to optimum hydrophilic–lipophilic balance, which is achieved by using hydrophilic and lipophilic surfactants in conjugation. Apart from this, mixed surfactants significantly lower the value of interfacial tension, thus, producing smaller droplet size and better stability [10]. Additionally, previous studies have also shown that mixed surfactant‐based nanoemulsions disperse readily into continuous phase which is desirable for colloidal based delivery system [9]. Keeping in view above mentioned benefits, mixed surfactants were used as a stabilizer in the preparation of LAA W/O nanoemulsions. In this study, we have used a combination of Tween 80 and soy lecithin as surfactants. Previously, we have successfully used this surfactant mixture for preparation of stable nanoemulsions.

LAA (Vitamin C) is a white, crystalline, ketolactone containing two hydroxyl groups which are ionisable [4, 11]. LAA performs numerous vital roles in our body such as collagen synthesis, activation of nervous system enzymes and increase the absorption of certain minerals and drug detoxification [12]. Apart from these, many health benefits are associated with ascorbic acid such as reduction in allergic reaction and steroids excretion [13]. Due to these favourable properties, it is used in food, pharmaceutical and cosmetic products. But unfortunately, it is lost in many food products during processing, storage and distribution due to oxidation [14]. Therefore, food industries are obsessed in fortification of their products with LAA because apart from fulfiling daily requirement of vitamin C, it also acts as antioxidant. But, it cannot be directly incorporated into different beverages and food products because a considerable amount of ascorbic acid is lost at room condition [15]. Thus, it should be protected from environmental conditions before its application into different food products. Hence, the purpose of the present research work is to incorporate the LAA in water‐in‐oil nanoemulsions. Previously, no study has been carried out on the mixed surfactant‐based LAA nanoemulsions.

The consumption of olive oil is increasing in the world due to its health effects. Olive oil contains higher proportion of unsaturated fatty acid and lower proportion of saturated fatty acid, which is beneficial for human health [16]. Additionally, olive oil is a rich source of phenolic compounds (simple phenols, secoiridoids and lignans), which have antioxidant properties [17]. Due to higher amount of antioxidants, olive oil lowers the incidence of diabetes mellitus, hypertension, cancer and coronary artery disease. Apart from this, olive oil also exhibit anti‐microbial and anti‐inflammatory properties [18]. Hence, olive oil is used in present study to take advantage of health benefits of olive oil.

Response surface methodology (RSM) is an effectual tool for investigating the relationship between multiple independent variables and responses for optimisation of products or processes [19]. This technique can be used for simultaneous optimisation of independent variables and their interaction for process optimisation, as well as product development [20, 21]. Previously, this technique had been used for various food products and processes for the optimisation of ingredient levels [22, 23]. During laboratory experiments, LAA nanoemulsions were affected by different independent variables (data not shown). Hence, there is need to optimise these variable. Optimum emulsifying conditions and interactive effect of these independent variables were determined using response surface methodology.

The purpose of this research work was to develop food grade LAA nanoemulsions and optimisation of preparation conditions through RSM in order to determine the influence of major independent variables on responses (nanoemulsions size and stability). Additionally, interactive effects of independent variables on response variables were also investigated through RSM. This study will have important implications in food industry to develop delivery system for food additives and nutraceutical components.

## 2 MATERIALS AND METHODS

### 2.1 Materials

Tween 80, soy lecithin (≥99% TLC) and MgSO_4_ (≥97%) were provided by Sigma‐Aldrich (St. Louis, MO, USA). LAA (>99%) was obtained from Tocris Bioscience (Bristol, UK). Refined olive oil was obtained from Punjab Oil Mills (Islamabad, Pakistan). Distilled and deionised water was used while performing experiments. Analytical grade reagents and solvents were used for experimentation.

### 2.2 Properties of nanoemulsion components

The constituents of nanoemulsion (olive oil, surfactants and LAA) were subjected to physicochemical analyses to investigate their role in nanoemulsions preparation and stability. The density of nanoemulsion components was determined using density metre (DS7800, KRUSS, Germany). Interfacial tension of olive oil (oil–water) was determined through the tensiometer (DSA100, KRUSS, Germany). The capillary viscometer (KV100, Brookfield, Middleboro, USA) was used to measure olive oil viscosity, and the rotational rheometer (RheolabQC, Antoon Par, Austria) was used to measure viscosity of lecithin and Tween 80 [24].

### 2.3 Nanoemulsions preparation

Initially, dispersed phase of water‐in‐oil nanoemulsions was formulated by dissolving 1% MgSO_4_ and LAA (0.5%–1.3%) in different amount of water (6%–14%). The continuous phase was comprised of refined, bleached and deodorized olive oil containing different concentrations of surfactants (Lecithin: Tween 80; 2:3). The continuous and disperse phases were mixed together, and the resulting premix was subjected to homogenisation (KRH‐I, KONMIX, Shanghai, China) to homogenise at 16,000 rpm for 15 min. After that, the size of coarse emulsions was further reduced using high‐pressure homogenisation (Microfluidics M‐110Y, Newton, USA) at 25°C and pre‐determined homogenisation pressure (80–160 MPa) for eight cycles.

### 2.4 Droplet size measurement

Droplet size was determined using a particle size analyser (Microtrac, Tri‐Blue, USA). During analysis, multiple scattering effects were avoided using 10% diluted nanoemulsion samples [23].

### 2.5 Evaluation of nanoemulsions stability

Droplet growth ratio was determined using previously reported method [7]. The growth ratio of LAA nanoemulsions was measured by calculating the difference in droplet size after 15 days with the zero day droplet size. Nanoemulsions stability was calculated by below‐mentioned formula.

Nanoemulsion growth ratio = droplet size after 15 days – droplet size at 0 days/Droplet size at 0 day.

### 2.6 Retention of l‐ascorbic acid

LAA concentrations in optimised nanoemulsions were calculated using Association of Official Analytical Chemists method No. 967.21 (2.6‐dichlorophenol indophenol titrimetric method) [25]. First, 1 g nanoemulsion was mixed with ethanol (10 ml) and sonicated for 20 min. After that, this mixture was centrifuged at 3000 rpm for 3 min. The resulting supernatant (1 ml) was used for the measurement of LAA concentration. Retention of LAA was determined using following equation:

$$R=\left(C_{LAA}/C_{LAA,0}\right)\times 100$$

Where *R* is LAA retention (%), C_LAA_ denotes the experimental value of LAA and C_LAA,0_ represents the initial concentration of LAA.

### 2.7 Experimental design

RSM was used to investigate the effect of water proportion (%), homogeniser pressure (MPa), amount of surfactants (%) and LAA contents on response variables (droplet size and stability of LAA nanoemulsions). The experimental design of independent variables is summarised in Table 1. Central composite design (CCD) along with quadratic model and 2^4^ factorial was used in this model. Each independent variable has five levels: ‐2, ‐1, 0, 1 and 2. Thirty treatments with six central points were formed. This research was carried out in randomised order. The coding of independent variables was carried out according to Equation (1).

(1) $$Y=Y_{0}–Y_{C}/\Delta Y$$

where *Y* denotes coded independent variable values, *Y*_0_ is actual variable values, *Y*_*C*_ represents central value and ∆*Y* is step change. Additionally, specific Equations (2), (3), (4), (5) for each independent variable are given below:

(2) $$y_{1}=\left(\text{W}\;-\;10\right)/2$$

(3) $$y_{2}=\left(\text{HP}\;-\;120\right)/20$$

(4) $$y_{3}=\left(\text{S}\;-\;8\right)/2$$

(5) $$y_{4}=\left(\text{A}\;-\;0.9\right)/\;0.2$$

where W, HP, S and A represents water proportion (%), homogenisation pressure (MPa), surfactant concentration (%) and LAA concentration (%), respectively.

**TABLE 1:** Independent variables and their corresponding levels for W/O nanoemulsion preparation

Independent variable	Symbol	Coded levels				
		−α	−1	0	+1	+α
Water proportion (%)	X_1_	6	8	10	12	14
Homogenisation pressure (MPa)	X_2_	80	100	120	140	160
Surfactant concentration (%)	X_3_	4	6	8	10	12
l‐ascorbic acid concentration (%)	X_4_	0.5	0.7	0.9	1.1	1.3

While designing these experiments, second‐order polynomial equation was used for response prediction as a function of emulsifying conditions. It is summarised in Equation (6).

(6) $$Y=\;\gamma_{0}+\;\gamma_{1}X_{1\;}+\;\gamma_{2}X_{2}+\;\gamma_{3}X_{3\;}+\;\gamma_{4}X_{4\;}+\gamma_{11}X_{1}^{2}+\gamma_{22}X_{2}^{2}\;+\gamma_{33}X_{3}^{2}\;+\;\gamma_{44}X_{4}^{2}+\;\gamma_{12}X_{1\;}X_{2}+\gamma_{13}X_{1\;}X_{3\;}+\;\gamma_{14}X_{1\;}X_{4\;}+\;\gamma_{23}X_{2\;}X_{3\;}+\;\gamma_{24}X_{2\;}X_{4\;}+\;\gamma_{34}X_{3\;}X_{4\;}$$

where *Y* indicates the responses, γ_0_ is constant, γ_i_ is linear term effects, γ_ii_ is squared effects, and γ_ij_ represents interactive effects. These coefficients were calculated through Software (Design Expert, Version 8.0.7.1).

### 2.8 Statistical analysis

Regressions analysis was performed on the data to fit second order polynomial equations for independent variables. The significance of these variables was checked through analysis of variance (ANOVA) by computing their *F*‐Value at the probability (*p*) of 0.05, 0.01 and 0.001. Response plots were generated to visualise the effects of preparation conditions on response values through Design Expert Software, Version 8.0.7.1.

## 3 RESULTS AND DISCUSSION

### 3.1 Fitting the models

The experimental and predicted values of response variables for LAA nanoemulsions are summarised in Table 2. A remarkable agreement was observed between experimental and predicted values, which are obtained from response surface methodology design. These experimental values were used to calculate the coefficients of polynomial equations for prediction response variables values. The following regression equations were obtained for the droplet size [Equation (7)] and emulsion stability [Equation (8)] by the application of RSM.

(7) $$\text{Droplet}\;\text{size}\;=\;+135.67-\;2.13X_{1\;}-\;5.54X_{2}-\;5.79X_{3\;}+5.12X_{4\;}+2.05X_{1}^{2}\;+2.44X_{1\;}X_{3\;}-\;2.69X_{1\;}X_{4\;}$$

(8) $$\text{Nanoemulsion}\;\text{stability}=\;+0.46-\;0.027X_{1\;}-\;0.021X_{2}-\;0.038X_{3\;}-0.025X_{4\;}\;+0.015X_{3}^{2}\;+\;0.014X_{4}^{2}-\;0.021X_{1\;}X_{3\;}$$

**TABLE 2:** Experimental design for l‐ascorbic acid nanoemulsions with independent variables, experimental and predicted values of responses

Run					Droplet size (nm)		Stability of emulsion	
	W (%)	P (MPa)	S (%)	A (%)	Experimental	Predicted	Experimental	Predicted
1	10	120	8	0.9	139	135.67	0.438	0.46
2	12	140	6	1.1	137	136.63	0.483	0.49
3	8	140	10	0.7	119	118.92	0.487	0.49
4	10	120	8	0.9	138	135.67	0.477	0.46
5	12	100	10	1.1	142	145.13	0.396	0.40
6	10	120	8	1.3	152	146.63	0.455	0.46
7	10	160	8	0.9	126	128.79	0.444	0.44
8	10	80	8	0.9	154	150.96	0.554	0.52
9	12	100	10	0.7	138	135.75	0.404	0.42
10	12	140	10	1.1	131	132.42	0.372	0.36
11	8	100	6	1.1	159	162.46	0.530	0.53
12	8	100	10	0.7	129	130.13	0.541	0.56
13	8	100	10	1.1	148	150.25	0.490	0.51
14	12	140	6	0.7	139	136.25	0.544	0.54
15	6	120	8	0.9	150	148.13	0.503	0.48
16	10	120	8	0.9	134	135.67	0.450	0.46
17	12	100	6	1.1	148	147.58	0.504	0.51
18	10	120	8	0.9	137	135.67	0.435	0.46
19	12	140	10	0.7	128	125.29	0.372	0.40
20	10	120	12	0.9	130	128.79	0.470	0.44
21	12	100	6	0.7	144	144.96	0.504	0.53
22	8	140	6	0.7	142	139.63	0.522	0.54
23	8	100	6	0.7	151	149.08	0.561	0.58
24	10	120	8	0.5	121	126.13	0.610	0.56
25	8	140	6	1.1	149	150.75	0.473	0.46
26	10	120	8	0.9	133	135.67	0.477	0.46
27	10	120	8	0.9	133	135.67	0.460	0.46
28	14	120	8	0.9	138	139.63	0.395	0.38
29	10	120	4	0.9	151	151.96	0.601	0.59
30	8	140	10	1.1	137	136.79	0.405	0.41

Abbreviations: A, l‐ascorbic acid concentration; P, homogenisation pressure; S, surfactant concentration; W, water proportion.

ANOVA results depicted that the representative models adequately represented experimental values. The coefficients of multiple determination (*R* ^2^) values of droplet size and emulsion stability for LAA nanoemulsions were 0.9375 and 0.9027, respectively (Table 3). The *R* ^2^ values for response variables were closer to unity, which indicates that the proposed model fitted well to actual data [7]. Lacks of fit values depicts the adequacy of proposed model [23].

**TABLE 3:** Significance of regression coefficients for l‐ascorbic acid nanoemulsion preparations

Variable	Droplet size of nanoemulsions (nm)		Stability of nanoemulsions	
	Regression coefficients	*p*‐value	Regression Coefficients	*p*‐value
*γ*_*0*_	135.67	0.0000	0.46	0.0000
*γ*_*1*_	−2.13	0.0088	−0.027	0.0002
*γ*_*2*_	−5.54	0.0000	−0.021	0.0019
*γ*_*3*_	−5.79	0.0000	−0.038	0.0000
*γ*_*4*_	5.12	0.0001	−0.025	0.0005
*γ*_*11*_	2.05	0.0072	−6.667	0.2185
*γ*_*22*_	1.05	0.1323	5.333	0.3204
*γ*_*33*_	1.18	0.0952	0.015	0.0114
*γ*_*44*_	0.18	0.7924	0.014	0.0153
*γ*_*12*_	0.19	0.8314	0.012	0.0886
*γ*_*13*_	2.44	0.0130	−0.021	0.0087
*γ*_*14*_	−2.69	0.0072	9.000	0.2052
*γ*_*23*_	−0.44	0.6205	−7.375	0.2949
*γ*_*24*_	−0.56	0.5255	−6.375	0.3630
*γ*_*34*_	1.69	0.0701	0.000	1.0000
*R* ^2^	0.9375		0.9027	

The analysis result has shown that there is non‐significant lack of fit in proposed model. The probability values of all parameters were less than 0.001 in present study, having non‐significant lack of fit. ANOVA was used to check coefficients significance. Greater *p*‐value, as well as smaller *F*‐value, stipulates non‐significant effects [26].

### 3.2 Properties of nanoemulsions components

The results regarding physico‐chemical analysis of nanoemulsion constituents are summarised in Table 4. LAA had higher density (1648 ± 1 kg m^−3^) as compared to water (1000 kg m^−3^). Additionally, the density of surfactants (soy lecithin and Tween 80) was higher as compared with LAA and olive oil, which had important effects on the nanoemulsions stability against gravitational separation. The rate of sedimentation of droplet was directly affected by density difference between continuous and dispersed phase [7, 27]. Nanoemulsion droplet size was depending on the interfacial tension between oil and water interface. When interfacial tension decreased, smaller droplets were produced [27]. The value of interfacial tension was relatively lower (33.7 ± 0.9 mN m^−1^) at olive oil–water interface. This interfacial value was reduced further by using mixed surfactants to achieve smaller particle size of nanoemulsions. When the viscosity ratio was closer to one, smaller size droplets were produced. The viscosities of olive oil (78.60 ± 1.1 mPa s) and water were significantly lower as compared with surfactants. The droplet size of nanoemulsions was also depended on the viscosity ratio of disperse to continuous phase [28].

**TABLE 4:** Physicochemical properties of w/o nanoemulsion components at ambient temperature

Components	Viscosity (mPa s)	Density (kg m^−3^)	Interfacial Tension (mN m^−1^)
l‐ascorbic acid	‐	1648 ± 1	‐
Olive oil	78.60 ± 1.1	915 ± 1	33.7 ± 0.9
Tween 80	373 ± 1.6	1088 ± 1	‐
Lecithin	8000 ± 3.5	1059 ± 1	‐

### 3.3 Influence of preparation conditions on responses

**FIGURE 1:**
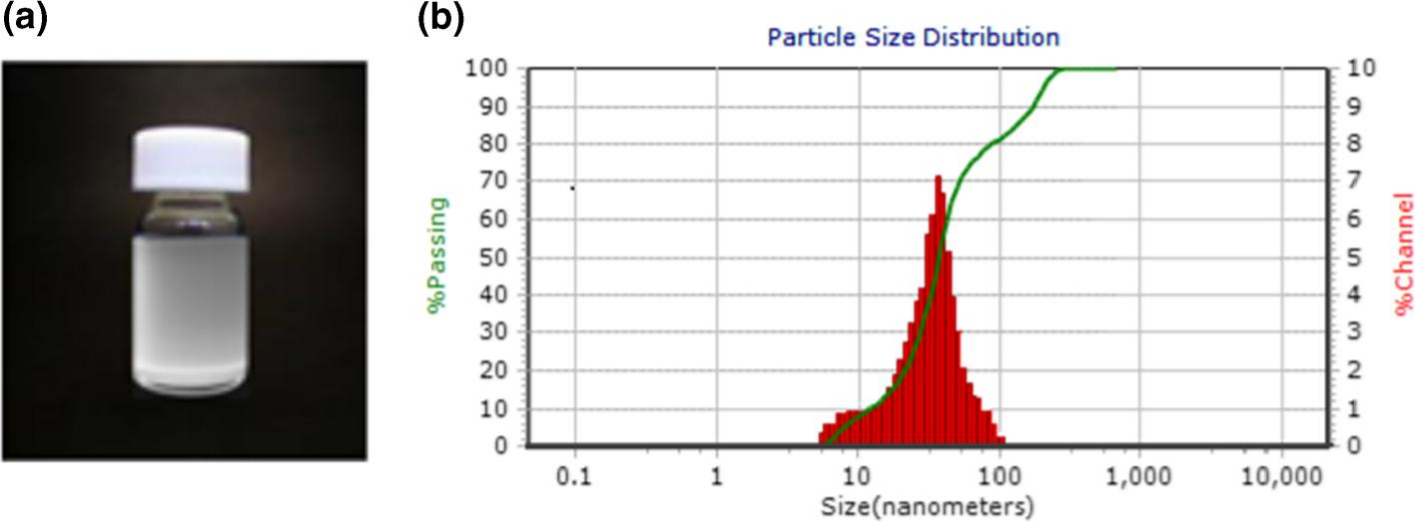
(a) Visual appearance of l‐ascorbic acid nanoemulsions. (b) Particle size distribution of l‐ascorbic acid nanoemulsions

Mixed surfactant‐based LAA nanoemulsions were prepared successfully using high pressure homogenising technique (Figure 1). The influence of preparation conditions on droplet size and nanoemulsion stability is given in Table 2. Additionally, regression coefficients values of different response variables are summarised in Table 3.

#### 3.3.1 Droplet size of l‐ascorbic acid nanoemulsions

**FIGURE 2:**
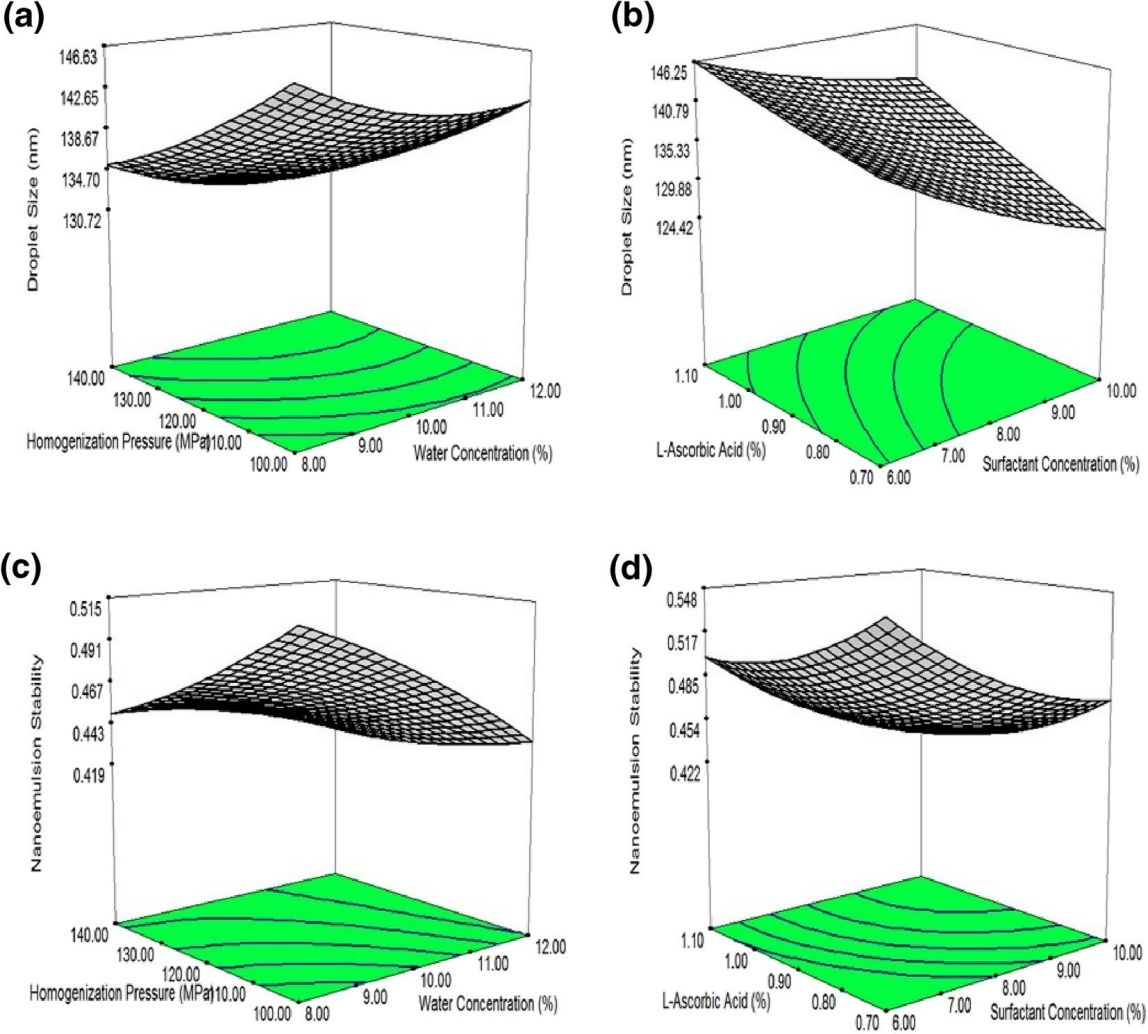
3D graphic surface optimisation of (a) droplet size (nm) versus homogenisation pressure (MPa) and water proportion (%), (b) droplet size (nm) versus surfactant concentration (%) and l‐ascorbic acid concentration (%), (c) stability of nanoemulsions versus homogenisation pressure (MPa) and water proportion (%) and (d) stability of nanoemulsions verses surfactant concentration (%) and l‐ascorbic acid concentration (%)

Droplet size in LAA nanoemulsion was predominantly depended on the water proportion because it significantly effects droplet size at liner (*p* < 0.01), quadratic (*p* < 0.05) and interactive terms with surfactant (*p* < 0.05) and LAA (*p* < 0.01). The size of LAA nanoemulsion droplets increased with the increase in water proportion (Figure 2a). Initially, smaller droplets were produced during high pressure homogenisation in the presence of enough surfactants. But, in the presence of higher proportion of disperse phase, enough emulsifier was not present in the chamber to hold newly formed droplets, which promote coalescence [24]. Other variables that have pronounced effects on droplet size of LAA nanoemulsions were linear terms of homogeniser pressure (*p* < 0.001), surfactant contents (*p* < 0.001) and LAA concentration (*p* < 0.001). The combined effects of homogeniser pressure and water proportion on droplet size of LAA nanoemulsions are illustrated in Figure 2a Water proportion and homogenisation pressure exert quadratic effect on the particle size of LAA nanoemulsions. The droplet size significantly reduced in the presence of higher homogenisation pressure if surfactant amount is enough to hold newly formed droplets [29]. Initially, smaller droplets of nanoemulsion were formed at lower water proportion but at higher concentration of dispersed phase, a significant increase in LAA nanoemulsion droplets was observed. These results were in agreement with the previous studies which were carried out on nanoemulsions [23, 24]. The interactive effects of LAA and surfactant concentration on size of droplets are given in Figure 2b. Both these variables exert linear effect on the droplet size of nanoemulsions. The droplet size of nanoemulsions had inverse relation with surfactant concentrations. When the concentration of the surfactants was increased, it will reduce interfacial tension which produces droplets having smaller size [30]. Linear relation was observed between the size of droplets and LAA concentration because higher concentration of LAA increases disperse phase viscosity. Due to increase in viscosity, more energy is required to disrupt larger droplets into smaller one [27, 28]. Similar finding was reported in previous research regarding the change of particle structure with the viscosity change [31].

#### 3.3.2 Nanoemulsion stability

Independent variable with greater effect on the stability of LAA nanoemulsion was concentration of surfactant as it has significant effects at linear (*p* < 0.001), quadratic (*p* < 0.05) and interactive level (*p* < 0.01) on the stability of nanoemulsions. Surfactants increase the stability of nanoemulsions by stabilising newly formed droplets. Hence, it prevents the coalescence and flocculation in nanoemulsions droplets [14]. However, excessive use of surfactant may negatively affect the stability of LAA nanoemulsions due to the movement of water between droplets by surfactant micelles, which have positive effect on particle growth [32]. The stability of nanoemulsions understudy was greater than nanoemulsions prepared with single surfactant [24].

The stability of nanoemulsions increased due to use of mixed surfactants, which significantly reduce interfacial tension and increase loading capacity of dispersed phase through the formation of intercalating structure at interface [33]. Other variables having pronounced effects on nanoemulsions stability were the linear effect of homogenisation pressure (*p* < 0.01), water proportion (*p* < 0.001), LAA concentration (*p* < 0.001) and quadratic effect of LAA concentration (*p* < 0.05).

The combined effects of water proportion and homogeniser pressure on nanoemulsion stability are illustrated in Figure 2c. These variables had linear effect on the stability of water‐in‐oil nanoemulsions. The stability of these nanoemulsions increased when higher homogenisation pressure was used for preparation of nanoemulsions. This stability was achieved due to smaller droplets having greater stability against coalescence, flocculation and aggregation [6]. Higher water proportion decreases the stability due to increase in interfacial tension value at interface of water/oil. Hence, larger size droplets of nanoemulsions were generated which negatively affect the stability of LAA nanoemulsions [8]. The combined effects of surfactant concentration and LAA on nanoemulsion stability are depicted in Figure 2d. These variables exert quadratic effects on LAA nanoemulsions stability. Surfactant decreases the value of interfacial tension at the interface of water and oil and increases the stability of LAA nanoemulsions [30]. The nanoemulsions stability was increased with the increase in LAA concentration due to their higher density than water because it decreases the difference of density between continuous and dispersed phase and stabilised the emulsion against gravitational separation [27]. During storage, stability of nanoemulsions decreases due to Ostwald ripening [2].

### 3.4 Optimisation of the l‐ascorbic acid W/O nanoemulsions

In order to visualise the effects of independent variables on responses, response surface graphs were generated through Design Expert Software. For optimising the preparation conditions for nanoemulsions, response surface graphs were generated for droplet size and nanoemulsions stability (Figure 2). These graphs were prepared by holding the values of two variables constant (central values) while varying the values of other two variables within experimental range. Figure 2a,b was generated by varying the proportion of disperse phase and homogenisation pressure at 8% surfactant concentration and 0.9% LAA. By keeping the values of water proportion at 10% and homogenisation pressure at 120 MPa, response plots (Figure 2c,d) were drawn by varying the concentrations of LAA and surfactants. Generally, these graphs demonstrated complex interactions between independent variables.

Numerical optimisation for LAA water‐in‐oil nanoemulsions was carried out through selecting desired goals. Desirability function was used to select the optimum conditions. The desired goals for LAA nanoemulsions were minimum water proportion, minimum homogenisation pressure, lower amount of surfactant and maximum LAA to achieve minimum droplet size and maximum LAA nanoemulsions stability. Ten different sets of emulsifying conditions (different desirability values) were obtained through numerical optimisation. The set of emulsifying conditions with maximum desirability value was picked as optimum conditions. The values of optimised condition at maximum desirability were 9.04% water proportion, 114.48 MPa homogenisation pressure, 7.36% surfactant concentration and 1.09% LAA concentration. The values of predicted responses at optimised conditions were 146.88 nm and 0.472 for droplet size and nanoemulsion stability, respectively.

### 3.5 Verifications of model

The desirability of model for the response values prediction was checked by performing experiments using optimum preparation conditions (11.35% water proportion, 136.08 MPa homogenisation pressure, 6.73% surfactant concentration and 0.78% LAA concentration). Nanoemulsions were prepared using optimum combination of independent variables to measure their response variables. The values of experimental and predicted responses were compared in order to validate RSM model. The predicted response values at selected preparation conditions were 146.88 nm and 0.472 for size of droplets and nanoemulsion stability, respectively. The experimental values of nanoemulsions droplet size and stability were 142.25 ± 1.9 nm and 0.479 ± 0.011, respectively (Table 5). The actual and predicted response values indicate no significant difference.

**TABLE 5:** Comparison of predicted and experimental values of droplet size and nanoemulsion stability at optimum emulsifying conditions

Optimum Conditions	Coded levels	Actual levels
Water proportion (%)	−0.48	9.04
Homogenisation pressure (MPa)	−0.28	114.48
Surfactant concentration (%)	−0.32	7.36
l‐ascorbic acid concentration (%)	+0.95	1.09

Responses	Predicted values	Experimental values
Droplet size (nm)	146.88	142.25 ± 1.9
Nanoemulsion stability	0.472	0.479 ± 0.011

### 3.6 l‐ascorbic acid retention

**FIGURE 3:**
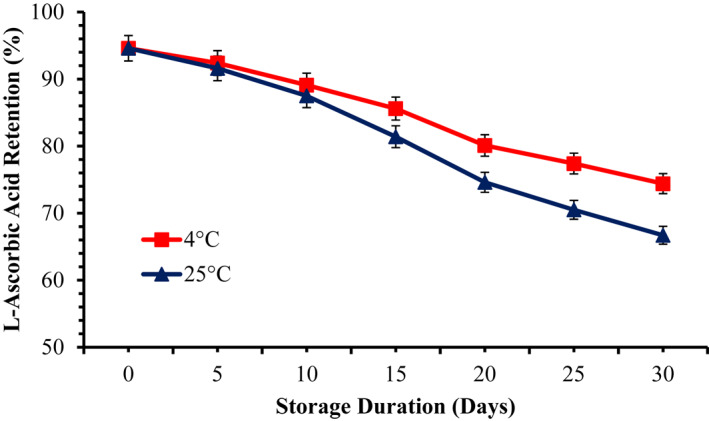
l‐ascorbic acid retention during one month storage at 4°C and 25°C

Nanoemulsions were prepared through optimised emulsifying conditions, and LAA retention was measured during 1 month storage at 4°C and 25°C. In general, degradation of LAA was observed during storage. The LAA retention in nanoemulsions during storage studies is given in Figure 3. Storage and temperature exert significant effect (*p* ≤ 0.05) on the retention of LAA during storage studies. At the end of 1‐month storage study, the retention of LAA in nanoemulsions store at 4°C and 25°C was 74.4% and 66.7%, respectively. Previous studies also reported major effect of temperature on LAA degradation [8, 11]. Light also increases the oxidative degradation of LAA [14]. Previous studies reported that retention of LAA was higher in emulsion‐based delivery system as compared with Milli‐Q water because degradation of LAA increased in aqueous solution due to rapid ionisation [34].

## 4 CONCLUSIONS

The present study was conducted to prepare LAA nanoemulsions and to optimise their preparation conditions using RSM. LAA was successfully incorporated into food grade nanoemulsions. RSM analysis depicted that response values can be predicted through second‐order polynomial model. Numerical optimisations of nanoemulsions were conducted using desirability function to obtain desirable characteristics. The optimum ingredients levels for LAA nanoemulsion preparation were 9.04% water proportion, 114.48 MPa homogenisation pressure, 7.36% surfactant concentration and 1.09% LAA concentration. Whereas, experimental values for droplet size and stability of nanoemulsion were 142.25 ± 1.9 nm and 0.479 ± 0.011, respectively. Additionally, the retention of LAA was higher at lower temperature and lower at higher temperature.
